# Diabetes‐Related Foot Disease as the Principal Reason for Hospital Admission: 5 Years of Data From a Major Tertiary Hospital in Aotearoa New Zealand

**DOI:** 10.1002/jfa2.70225

**Published:** 2026-10-17

**Authors:** Michele Garrett, Rinki Murphy, Arier Lee, Peter A. Lazzarini, Timothy Kenealy

**Affiliations:** ^1^ Faculty of Medical and Health Sciences School of Medicine University of Auckland Auckland New Zealand; ^2^ Te Toka Tumai Auckland Te Whatu Ora | Health New Zealand Auckland New Zealand; ^3^ Counties Manukau Te Whatu Ora | Health New Zealand Auckland New Zealand; ^4^ Department of Epidemiology and Biostatistics School of Population Health University of Auckland Auckland New Zealand; ^5^ Australian Centre for Health Services Innovation School of Public Health and Social Work Queensland University of Technology Brisbane Australia; ^6^ Allied Health Research Collaborative The Prince Charles Hospital Brisbane Australia; ^7^ Department of General Practice & Primary Health Care School of Population Health University of Auckland Auckland New Zealand

**Keywords:** amputation, diabetes foot disease, hospitalisations, Māori, New Zealand

## Abstract

**Objective:**

To examine hospitalisations caused by diabetes‐related foot disease (DFD) at the largest tertiary hospital in Aotearoa New Zealand.

**Methods:**

We analysed hospital admission data for DFD‐caused hospitalisations from Auckland City Hospital (ACH), from 1 July 2014 to 30 June 2019, for people 15 years or older, using ICD10‐AM discharge codes and amputation procedure codes. DFD‐caused hospitalisations were defined where DFD was the principal reason for admission. Demographic variables and length of stay were also collected.

**Results:**

During the study period, out of the total 11,222 hospitalisations in persons with diabetes, 1605 (14.3%) were DFD‐caused hospitalisations, which included 265 (16.5%) minor amputations and 94 (5.9%) major amputations. There were a total of 71,033 hospital bed days in persons with diabetes, of which 16,992 (23.9%) hospital bed days were due to DFD. Median length of stay in days was 4 (IQR: 1–8) for persons with diabetes; 7 (3–14) for DFD‐caused hospitalisations, 13 (8–22) for minors and 17 (13–26) for major amputations. The DFD‐caused hospitalisation rate per 1000 persons with diabetes increased by 86% from 6.2 (95% CI: 5.2, 7.2) in 2014/15 to 11.5 (10.2, 12.8) in 2018/19 and combined minor and major amputation rates increased 82% from 1.1 (0.7, 1.5) to 2.0 (1.4, 2.5). The rate of first DFD‐caused hospitalisation was highest for Māori at 7.5 (5.8 9.5), followed by Pacific at 5.3 (4.5, 6.2) and lowest for European/Other at 3.7 (3.3, 4.1) per 1000 person‐years with diabetes.

**Conclusion:**

DFD‐caused hospitalisations and amputations made up nearly a quarter of all hospital bed days used by people with diabetes and nearly doubled over 5 years. The highest rates were found in adults of Māori and Pacific ethnicity. Most DFD‐caused hospitalisations did not involve amputations (78%). The high and increasing burden of DFD‐caused hospitalisations suggest surveillance could monitor necessary changes to preventative foot care services.

## 1 Introduction

Diabetes has reached pandemic proportions worldwide [1], affecting 9.3% of adults and is projected to rise by around 20% by 2045, principally due to type 2 diabetes (T2DM) [2]. In Aotearoa New Zealand (Aotearoa), T2DM affects approximately 90% of adults with diabetes and is forecasted to rise by up to 90% by 2045 [3, 4]. Furthermore, rates of diabetes in Aotearoa vary considerably by ethnicity and are higher among Māori, Pacific and South Asian ethnicities compared with European/Other ethnicities [5]. People with diabetes are at a high risk of developing complications, including cardiovascular, renal, eye and foot disease [6]. Among these, diabetes‐related foot disease (DFD), including amputations [7], are considered amongst the most burdensome yet avoidable complications of diabetes [8].

Worldwide, DFD is the leading cause of amputations and diabetes‐related hospitalisations [9, 10]. Amputations are considered avoidable for most people with diabetes if using best‐practice foot care. Hence, amputation rates have been widely used as a national quality indicator for diabetes and DFD [11, 12, 13, 14, 15]. However, amputations are now performed in fewer than 30% of DFD‐caused hospitalisations [10, 16]. DFD‐caused hospitalisations typically result in higher hospital utilisation due to longer stays and more procedures compared with other diabetes‐related hospitalisations [8]. The risk of DFD‐caused hospitalisation and amputation increases with longer diabetes duration, persistent hyperglycaemia, infection and peripheral artery disease [17] and can vary by age, gender, ethnicity, socioeconomic deprivation and geographical location [18].

In Aotearoa, diabetes is also the leading cause of amputations, with the highest rates among Māori [19, 20] and increasing among Pacific peoples in the Northern Region of Aotearoa [21]. Furthermore, the Te Tāhū Hauora (Health Quality and Safety Commission) in Aotearoa has introduced national indicators to promote quality improvement in diabetes care, including monitoring the proportion of total hospitalisations and amputations that occur in people with diabetes [22]. Therefore, DFD‐caused hospitalisations, the leading cause of hospitalisations in people with diabetes, alongside amputations, could serve as important quality indicators for diabetes and DFD care in Aotearoa and worldwide [7, 10].

Although studies have examined amputation rates in Aotearoa [19, 20], only one 1998 study has reported rates of DFD‐caused hospitalisations [23]. This study found that the national incidence of DFD‐caused hospitalisations increased by 90% from 13.6 in 1980 to 25.8 per 100,000 resident population in 1993. The study also estimated the total inpatient cost to be $NZ10‐11 million annually [23]. However, to the best of our knowledge, no further study has investigated DFD‐caused hospitalisations in Aotearoa.

Understanding the burden of DFD‐caused hospitalisations within a major tertiary referral service may help address this evidence gap. Situated in the Northern Region of Aotearoa, Auckland City Hospital (ACH) is the country's largest tertiary hospital, with approximately 1175 beds, including 800 adult medical and surgical beds. ACH provides regional vascular services and performs most amputations for Auckland, Waitematā, and Te Tai Tokerau districts (District Health Boards). Delivering specialist vascular care across this large geographical area can present challenges, particularly for people living north of the city, where travel distances to ACH can exceed 350 km. In 2018, approximately 55,978 people were living with diabetes across these three districts. Collectively, the districts had a population of 1,294,200 people, representing approximately 27.5% of New Zealand's total resident population of 4,699,755 [24, 25].

Therefore, we examined trends in DFD‐caused hospitalisations among adult residents living in the Auckland district and the broader ACH catchment area. Findings should shed new light on the burden to the health system caused by DFD‐caused hospitalisations and help indicate the quality, effectiveness, and equity of DFD services in Aotearoa.

## 2 Methods

### 2.1 Setting

We analysed hospital admissions to ACH between 1 July 2014 to 30 June 2019 for individuals aged 15 years and older. Age ≥ 15 years was chosen as diabetes care in Aotearoa transfers to adult services at 15 years old and hence all those ≥ 15 years would be admitted with diabetes to ACH [26]. Less complex DFD hospitalisations admitted to hospitals in Waitematā and Te Tai Tokerau districts were not captured in this study.

### 2.2 Data Sources

Three data sources were used for this study: the ACH hospital administrative health database, the Virtual Diabetes Register (VDR), which is the Aotearoa national Ministry of Health's database for diabetes populations and used in studies for population estimates [4, 27], and the Statistics New Zealand estimated resident population database [25].

Data were analysed in two populations: hospital admissions for people from any district regardless of residential address, and admissions from residents of the Auckland district only. Hospitalisations of Auckland district residents were used to estimate crude and standardised DFD hospitalisation rates in the Auckland district population.

### 2.3 Hospitalisations

Hospital discharge codes were used to identify all included hospitalisations in people with diabetes and DFD‐caused hospitalisations. These codes are recorded for every hospital admission in ACH using the International Statistical Classification of Diseases and Related Health Problems, 10th Revision, Australian Modification (ICD‐10‐AM). Diabetes and DFD were identified using standard sets of ICD‐10‐AM diagnosis codes that are typically used for diabetes and DFD in other studies [10, 28] or amputation procedures identified using standard procedure codes in the Australian Classification of Health Interventions procedure codes (see Supporting Information S1).

Hospitalisations in persons with diabetes were classified if a diabetes diagnosis code was listed as a reason (any listed code) for the hospital admission. DFD‐caused hospitalisations were classified if a DFD code was the principal reason (the first listed code) for the hospital admission, or an amputation procedure code was listed in a hospitalisation with a corresponding diabetes code, as used in other similar studies [7, 10, 29, 30, 31]. Amputation procedures were grouped into major (above the ankle) or minor (at or distal to the ankle) amputations [7]. Only the highest (most proximal) level of amputation per admission was reported when describing hospitalisations. All amputations are included to describe the numbers and trends of amputations. Diabetes confined to pregnancy was excluded.

### 2.4 Variables

Variables of interest collected for each included hospitalisation were gender, age at admission, ethnicity, admission and discharge date, length of stay (LoS) in bed days and district of residence. Gender was categorised as male or female, excluding other gender designations due to small numbers and re‐identification risks. Age at admission was collected in whole years [26]. Reported prioritised ethnicity [32] was used and grouped into Māori, Pacific, Indian, and European/Other as per other NZ studies [19]. The district of residence was the place of usual residence for an individual (which might not be the district providing hospital services). LoS was defined as days from the admission date to the discharge date. If a subsequent admission occurred on the same day or the following day, this was counted as a single admission and the summed LoS was recorded as the LoS for that admission.

### 2.5 Statistical Analysis

Descriptive statistics were used, as appropriate, to summarise the data. Counts and proportions were used to present data for admissions in people with diabetes, DFD‐caused hospitalisations and amputations stratified by gender, age and ethnicity. To analyse trends over time for rates and counts, we employed annual percentage change and Poisson regression models.

Age standardised incidence rates for DFD‐caused hospitalisation in the Auckland resident population were calculated using the Auckland district resident structure in 2018 from the district estimated population data. Crude rates in the diabetes population in the Auckland district were calculated from the national Ministry of Health, Virtual Diabetes Register (VDR) for ages 15 years or older [27]. Admission rates allowed each person to contribute more than one admission. Age standardised rates and crude rates were calculated using age at first DFD hospitalisation during the 5‐year study period, meaning that each person could be counted only once. A *p* value < 0.05 was considered statistically significant.

Statistical analyses were performed using SAS statistical software, version 9.4 (SAS Institute Inc., Cary, NC) and Stata statistical software, version 13.1 (College Station, TX). We used Julius AI (Julius AI Inc., 2024), a GPT‐4‐powered artificial intelligence assistant specialised in data science and statistical analysis to assist with linear regression analysis and data visualisation [33] and checked using Stata.

Due to privacy concerns, the datasets used in this study are not publicly accessible. Ethics was granted from the hospital ethics committee (#A+8723).

## 3 Results

### 3.1 Characteristics of DFD‐Caused Hospitalisations in Auckland City Hospital and for Auckland District Resident Population

Table 1 displays the characteristics of the 11,222 hospitalisations from 4944 people with diabetes hospitalised from any district over the 5‐year period, of which 1605 (14.3%) were DFD‐caused hospitalisations, 266 (2.4%) and 102 (0.9%) with minor and major amputation procedures. One minor and eight major amputations occurred during a hospitalisation without a coincident DFD ICD‐10‐AM code. There were a total of 71,033 bed days used for the 11,222 hospitalisations in people with diabetes, of which DFD‐caused hospitalisations accounted for 16,992 (23.9%). Of the 1605 DFD‐caused hospitalisations, the mean age was 66 (SD 13.8) years, 68% were male, and 42% were of Māori or Pacific ethnicity. Median length of stay was 4 (IQR:1–8) days for persons with diabetes, 7 (3–14) for DFD‐caused hospitalisations (including amputation), 13 (8–22) for those having minor and 17 (13–26) days for major amputation. DFD‐caused hospitalisations were younger, had more males, and more Māori and European/other ethnicities than hospitalisations with diabetes (all, *p* < 0.001).

**TABLE 1:** Characteristics of hospitalisations with diabetes, caused by diabetes foot disease (DFD‐caused), minor and major amputations, at Auckland City Hospital over 5 years (2014/15 to 2018/19), and Auckland district residents only.

Variables	Hospitalisations with diabetes		DFD‐caused hospitalisation^b^		Minor amputation^c^		Major amputation		*p*‐value^e^
	ACH	Auckland district	ACH	Auckland district	ACH	Auckland district	ACH	Auckland district	
Number	11,222	9157	1605	1076	266	134	102	48	
Gender^a^									< 0.001
Male	6598 (59.8%)	5253 (57.4%)	1094 (68.2%)	710 (66.0%)	202 (75.5%)	96 (71.6%)	66 (64.7%)	30 (62.5%)	
Female	4624 (40.2%)	3904 (42.6%)	511 (31.8%)	366 (34.0%)	64 (24.5%)	38 (28.4%)	36 (35.3%)	18 (37.5%)	
Age‐mean (SD)	67.8 (14.2)	68.7 (14.1)	66.6 (13.8)	67.1 (14.0)	66.7 (11.8)	66.3 (14.1)	68.8 (11.8)	67.1 (12.3)	< 0.001
Ethnicity^a^									< 0.001
Māori	1405 (12.5%)	987 (10.8%)	273 (17.0%)	152 (14.1%)	41 (15.4%)	14 (10.4%)	26 (25.5%)	8 (16.7%)	
Pacific	3555 (31.7%)	3052 (33.3%)	401 (25.0%)	305 (28.3%)	58 (21.8%)	36 (26.9%)	21 (20.6%)	16 (33.3%)	
Indian	1129 (10.1%)	966 (10.5%)	104 (6.5%)	79 (7.3%)	15 (5.6%)	8 (6.0%)	8 (7.8%)	5 (10.4%)	
European/other	5133 (45.7%)	4152 (45.3%)	827 (51.5%)	540 (50.2%)	152 (57.1%)	76 (56.7%)	47 (46.1%)	19 (39.6%)	
Hospital bed days	71,033 (100)	55,626 (100)	16,992 (23.9%)	10,433 (18.76%)	4396 (NA)	2012 (NA)	2130 (NA)	1142 (NA)	
Length of stay^d^	4 (1–8)	3.00 (1–8)	7 (3–14)	6 (3–13)	13 (8–22)	11.5 (7–22)	17 (13–26)	17 (14–29)	

^a^ Results are *n* (column section %).

^b^ Including amputation.

^c^ Excluding major amputation.

^d^ Results are median, (IQR).

^e^ *p*‐values compare ‘hospitalisations with diabetes’ with ‘DFD‐caused hospitalisations’.

There were 9157 hospitalisations of 3648 people with diabetes who were residents of the Auckland district. Of these 1076 (11.8%) were DFD‐caused hospitalisations, which occurred in 541 individuals. There were 134 (1.5%) minor amputation and 48 (0.5%) major amputation procedures. One minor amputation occurred during a hospitalisation without a coincident acute foot ICD‐10‐AM code.

### 3.2 Rates of DFD‐Caused Hospitalisations

Table 2 displays the hospitalisation and population numbers and rates per diabetes and resident population by financial year within Auckland district residents. Counts increased 100% for all DFD‐caused hospitalisations, 250% for major amputations, 68.2% for minor amputations and a 96% increase for combined minor and major amputations. The underlying population increases were modest, with the diabetes population increasing by 7.6% and the resident population by 10.3%.

**TABLE 2:** Numbers and incidence rates of DFD‐caused hospitalisations and amputations per 1000 persons with diabetes and 100,000 residents from Auckland district population 15+ years of age.

Financial year	2014/15	2015/16	2016/17	2017/18	2018/19	Total	Percentage	*p* value^
							Change	
Numbers								
Hospitalisations with diabetes	1472	1622	1889	2009	2165	9157	41.7	< 0.05
DFD‐caused hospitalisations	149	179	228	222	298	1076	100	< 0.05
Minor amputations	22	27	26	22	37	134	68.2	0.24
Major amputations	4	7	9	14	14	48	250	< 0.05
Combined amputations	26	34	35	36	51	182	96.2	< 0.05
Diabetes population	24,126	24,513	24,596	24,895	25,954	124,08	7.6	< 0.05
Resident population	326,921	331,659	337,714	346,621	360,503	1,703,418	10.3	< 0.05
Incidence per 1000 persons with diabetes (95% CI)								
DFD‐caused hospitalisations	6.2 (5.2, 7.2)	7.3 (6.2, 8.4)	9.3 (8.1, 10.5)	8.9 (7.8, 10.1)	11.5 (10.2, 12.8)	8.7 (8.2, 9.2)	85.8	< 0.05
Amputations	1.1 (0.7, 1.5)	1.4 (0.9–1.9)	1.4 (0.9, 1.9)	1.5 (0.9, 1.9)	1.97 (1.4, 2.5)	1.5 (1.2, 1.7)	82.4	< 0.05
Incidence per 100,000 residents (95% CI)								
DFD‐caused hospitalisations	46 (39, 53)	54 (46, 53)	68 (59, 76)	64 (56, 73)	83 (74, 92)	63 (59, 67)	80.4	< 0.05
Amputations	8 (5,11)	10 (7,14)	10 (7, 14)	10 (7, 14)	14 (10, 18)	11 (9, 12)	75.0	0.06

Within the diabetes population, incidence rates increased significantly over the 5‐year period by 86% for DFD‐caused hospitalisations from 6.2 (95% CI: 5.2, 7.2) in 2014/15 to 11.5 (10.2, 12.8) in 208/19 per 1000 person‐years with diabetes ≥ 15‐years (*p* = 0.013), and total amputation rates increased by 82% from 1.1 (0.7, 1.5) to 2.0 (1.4, 2.5) (*p* = 0.03). Within the resident population, incidence rates increased significantly by 80.4% for DFD‐caused hospitalisations from 46 (39, 53) in 2014/15 to 83 (74, 92) in 2018/19 per 100,000 person‐years ≥ 15‐years (*p* = 0.017), and total amputation rates increased by 75% from 8 (5, 11) to 14 (10, 18) (*p* = 0.058).

### 3.3 Rates of First DFD‐Caused Hospitalisations in the Auckland District Population

The age‐standardised rates of individuals at the age of their first DFD‐caused hospitalisation during the follow‐up period were 23.3 per 100,000 person‐years for females and 44.0 per 100,000 person‐years for males, with an age‐gender standardised rate of 33.5 per 100,000 person‐years.

Table 3 display rates of first DFD‐caused hospitalisations per 100,000 person‐years population ≥ 15‐year, by age group, and gender. The incidence rates of individuals at the age of their first DFD‐caused hospitalisation during the follow‐up period were 26.2 (95% CI: 24, 28.5) per 100,000 person‐years ≥ 15‐years, including 33.9 (30.3, 37.5) for males, 1.8 (95% CI: 1.4, 2.3) times higher than for females 1.9 (1.6, 2.2) per 100,000 person‐years. Incidence rates were higher in older age groups, ranging from 0.3 (0.2, 0.5) for 15–44 years to 17.0 (14.7, 19.5) for 75+ years age group.

**TABLE 3:** DFD Hospitalisation rate per 100,000 person years by ethnicity first admission age, gender, Auckland district population 15+ years of age.

Age group (years)	15–44	45–54	55–64	65–74	75+	Total	*p*‐value
Number	40	59	116	127	199	541	
All (95% CI)	3.3 (2.4, 4.5)	70 (53.3, 90.3)	45 (37.2, 54)	78.4 (65.3, 93.2)	170.1 (147.3, 195.5)	26.2 (24, 28.5)	
Gender							
Male (95% CI)	4.1 (2.5, 5.7)	21.8 (14.5, 29.2)	60.5 (46.9, 74.1)	115.5 (91.8, 139.2)	240.5 (197.5, 283.5)	33.9 (30.3, 37.5)	< 0.005
Female (95% CI)	2.5 (1.2, 3.8)	15.3 (9.3, 21.4)	30.3 (20.9, 39.6)	43.2 (29.1, 57.3)	117.8 (91.8, 139.2)	18.7 (16.1, 21.3)	

Table 4 displays the rates of first DFD‐caused hospitalisations per 1000 person‐year with diabetes ≥ 15‐years by ethnicity. Indian ethnicity was combined with European/Other ethnicity grouping due to the small number of DFD‐caused hospitalisations. The incidence rates of first DFD‐caused hospitalisation were highest in Māori at 7.5 (5.8, 9.5), followed by Pacific Peoples at 5.3 (4.5, 6.2), and lowest in European/Other at 3.7 (3.3, 4.1) per 1000 person‐years with diabetes ≥ 15‐years. Both Māori (Rates ratio 2 (95% CI: 1.6, 2.7)) and Pacific (Rates ratio 1.4 (1.2, 1.7)) have significantly higher rates of incident DFD‐caused hospitalisations compared to European/Other.

**TABLE 4:** DFD hospitalisation rate per 1000 person years with diabetes by first admission age and ethnicity, Auckland district population 15+ years of age.

Age group (years)	15–44	45–54	55–64	65–74	75+	Total	*p*‐value
Number	40	59	116	127	199	541	
All (95% CI)	2.5 (1.8, 3.4)	2.8 (2.1, 3.6)	3.6 (2.9, 4.3)	4.3 (3.6, 5.1)	9.2 (7.9, 10.6)	4.3 (3.9, 4.7)	
Ethnicity							
Māori (95% CI)	5.1 (2.3, 9.6)	8.2 (4.6, 13.5)	8.8 (5.6, 13)	6.8 (3.6, 11.6)	8.5 (3.7, 16.8)	7.5 (5.8, 9.5)	< 0.005
Pacific (95% CI)	4.8 (3.1, 7.2)	3.8 (2.5, 5.5)	4.8 (3.5, 6.6)	6.0 (4.2, 8.2)	8.4 (5.7, 11.9)	5.3 (4.5, 6.2)	< 0.005
European/other (95% CI)	0.7 (0.3, 1.5)	1.4 (0.8, 2.2)	2.4 (1.8, 3.1)	3.5 (2.8, 4.4)	8.0 (6.8, 9.4)	3.7 (3.3, 4.1)	

The differences in rates by ethnicity persisted across all age groups and are highest in the younger age groups. For Māori the highest difference is in the 15–44 age group RR = 6.8 (95% CI: 2.6, 18.4), with the difference continuing in the 45–54 age group RR = 5.7 (2.9, 11.4) and then narrowing with increasing age. Pacific had a similar pattern to Māori in the youngest age group (RR = 6.5, CI: 2.8, 15.1) but showed a more rapid decrease in differences with increasing age. By age 75+ rates are similar across all ethnicities as illustrated in Figure 1.

**FIGURE 1:**
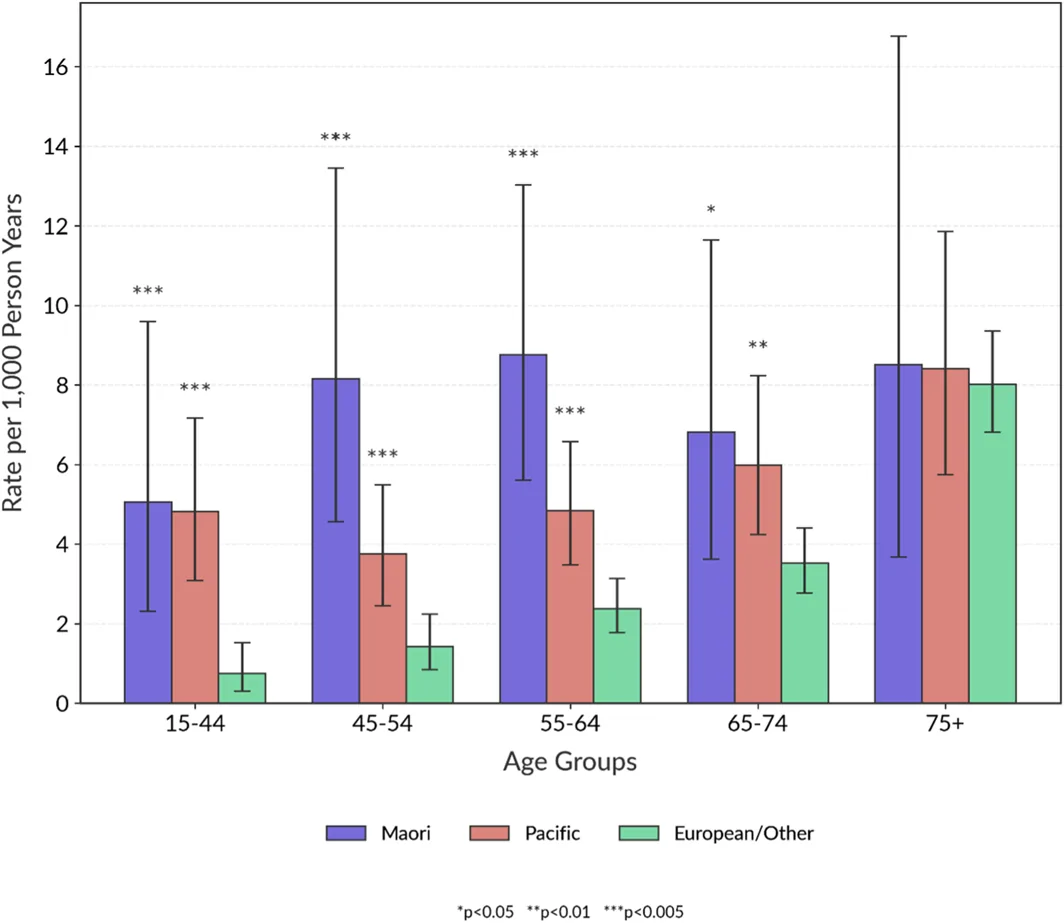
First DFD‐caused hospitalisation rates per 1000 person years with diabetes by ethnicity and age group: Auckland district diabetes population.

## 4 Discussion

This study is the first in 30 years to investigate trends in DFD‐caused hospitalisations in Aotearoa, revealing an increasing burden of DFD hospitalisations alongside the complex trends in amputation rates, and highlighting persistent ethnic differences in DFD outcomes. There were several important findings. First, we observed an increasing trend in DFD‐caused hospitalisations over the period by approximately 100% accompanied by concerning increases in both major (250%) and minor (68%) amputations. Correspondingly, rates of DFD‐caused hospitalisations increased significantly in the diabetes and resident populations. Second, DFD‐caused hospitalisation represented a substantial burden accounting for 14% of hospital admissions and 24% of bed days for those with diabetes, with increasing median lengths of stay for DFD‐caused hospitalisations (7 days), minor (13 days) and major amputations (17 days), compared to those hospitalised with diabetes (4 days). Third, we found ethnic disparities in the incidence of DFD‐caused hospitalisations, with the highest rates observed in Māori at 7.5 per 1000 person‐years, followed by Pacific people at 5.3 per 1000 person‐years, and the lowest for Europeans/Others at 3.7 per 1000 person‐years within the Auckland district. Lastly, males, and those aged 75+, had the highest rates of incident DFD‐caused hospitalisations though concerningly DFD‐caused hospitalisations and amputations occurred for people aged 15–44 years with a high rate of DFD‐caused hospitalisations in the 45–54 age group, 70 (95% CI: 53.3, 90.3) per 100,000 person‐years ≥ 15‐years.

Our results indicate a significant increasing burden of DFD hospitalisations at ACH over a recent 5‐year period with a 15.4% annual increase, a trend that mirrors increasing hospitalisations for other diabetes‐related complications in Aotearoa [34]. Previous research in Aotearoa reported a 90% increase in DFD hospitalisations for the total population over a 13 year period (1980–1993) from 13.56 per 100,000 total population to 25.79 [23]. Although our current reported rate of 63 per 100,000 is 2.4 times higher than the rate reported in 1993, it is lower than 91.7 per 100,000, the projected 5% annual increase from 1993. The lower‐than‐expected trend may reflect improvements in care pathways, structured diabetes foot programmes, and the progressive implementation of national quality standards since the 1990s [35]. Our observed annual increase of 8.4% in recent years indicates a concerning acceleration in incidence rates.

The 85% increase in rates of DFD‐caused hospitalisations for the diabetes population in the Auckland district aligns with trends observed in Australian studies [10, 31, 36], although direct comparisons with these studies are limited by differences in case definitions, denominator populations, and follow‐up protocols. In contrast, a review of 21st century studies reported non‐significant decreasing rates in some settings, which may reflect differences in the denominator population and earlier study periods [10]. The overall incidence of first DFD‐caused hospitalisations in our study (4.34 per 1000 person‐years) was in concordance with the Fremantle, Australia study (4.5 per 1000 person‐years), suggesting similar disease burden despite methodological differences [36]. Collectively, our findings provide a contemporary snapshot of DFD hospitalisation burden at ACH and support reporting DFD hospitalisations rates alongside amputation rates [10]. The substantial rise in DFD‐caused hospitalisations suggests factors beyond increasing prevalence may be contributing, including more severe DFD, changes in diabetes care and foot services, or more aggressive disease at an earlier age of onset. Given the preventable nature of DFD and its significant contribution to health care utilisation, the increasing rate of DFD‐caused hospitalisations highlights a growing burden on the health system and reinforces the need for strengthened prevention strategies. A standardised national measure of DFD‐caused hospitalisation rates could provide a valuable health system performance indicator for monitoring trends and evaluating the effectiveness of preventive foot care services.

While major amputations remained uncommon (0.06%–1.24% of admissions), the 250% relative increase observed over the study period is concerning and contrasts with reported declining trends internationally. Our observed rise in minor amputations adds to the inconsistent trends in minor amputations reported by Lazzarini et al. [10] Aotearoa has a national estimated major amputation rate of 6.4 per 100,000 total population, which is double the rate for the United Kingdom and approximately 1.67 times the rate for Australia [37, 38]. Although the number of major amputations reported in this study was small and should be interrupted cautiously, the upward trend warrants attention given Aotearoa's already high amputation rate. Consistent with other studies [10, 16], only 22.3% of DFD hospitalisations resulted in any amputation (16.5% minor and 5.8% major), indicating that most DFD‐caused hospitalisations do not lead to amputation. These findings suggest that amputation rates alone underestimate the burden of DFD and support the inclusion of DFD hospitalisations alongside major and minor amputations when evaluating service performance and outcomes.

DFD‐caused hospitalisation at ACH accounted for 14.3% of admissions among people with diabetes, substantially higher than the 2.0%–8.7% reported in Australian studies [39, 40, 41]. Similarly, a systematic review and meta‐analysis reported a pooled prevalence of DFD of 4.7% among general inpatient populations [42]. Our reported higher proportion of DFD‐caused hospitalisation at ACH may reflect its role as a tertiary referral centre managing complex cases. DFD also accounted for a disproportionate share of hospital resources, occupying nearly a quarter of total bed days among people with diabetes, consistent with other studies [8, 29] and supports the notion that DFD is a leading cause of diabetes‐related hospitalisations [10]. Furthermore, patients admitted with DFD had a mean length of stay 1.8 times longer than that of people admitted with diabetes for any cause, highlighting the substantial impact of diabetes‐related foot ulcers (DFU) and their complications on healthcare utilisation [8, 39].

DFD hospitalisations incidence rates among Māori and Pacific were 2 and 1.4 times higher, respectively, than non‐Māori relative to the total adult and adult diabetes population, consistent with previous studies of amputation rates [19, 20]. Those studies reported similar or lower amputation rates for Pacific Peoples than non‐Māori [20], contrasting with our findings. However, a recent audit from the same districts reported higher amputation rates among Pacific peoples than non‐Māori [21], suggesting an increasing incidence of DFD for Pacific peoples in Auckland. Health disparities in diabetes‐related complications, including DFD, have been widely reported among Indigenous and ethnic minority populations [43, 44, 45]. In Aotearoa, health inequities experienced by Māori and Pacific are linked to systemic racism, distrust and lower satisfaction in the health system, and the ongoing effects of colonisation compounding effects for Māori [46, 47, 48]. These factors may contribute to barriers to timely, culturally appropriate preventive care, delaying diagnosis and management of DFD [49], particularly culturally relevant podiatry services to meet the needs of Māori [50, 51]. Our findings reinforce the need for culturally safe interventions and equity focused DFD services to improve outcomes.

Previous studies have consistently reported a greater prevalence of DFD complications and amputations in males compared to females [20, 52, 53]. Consistent with these findings, males in our study experienced 1.8 times more DFD hospitalisations than females, with age‐standardised rates of 3.39 and 1.87 per 10,000 person‐years, respectively. These differences may reflect variations in underlying risk factors, access to healthcare, health‐seeking behaviours, and adherence to treatment [18]. Notably, the diabetes prevalence in Aotearoa was only 1.1 times higher in males than in females (41.4 vs. 3.52 per 1000 population) [54].

Whilst the risk of DFD and amputation generally increases with age, potentially due to diabetes duration and the heightened risk of age‐related complications [18, 55, 56], our study observed several hospitalisations and amputations in individuals younger than 45 years. Similar findings have been reported in Australia, where DFD hospitalisation rates have increased more rapidly in younger age groups [31, 36]. The Fremantle Australia study reported the highest incidence rate of DFU‐related hospitalisation in the youngest age group (31–40‐years old) [36]. Several factors may contribute to this trend. The prevalence of T2DM is increasing in younger populations [57, 58], including in Aotearoa [59, 60, 61]. Earlier onset T2DM may follow a more aggressive disease course and faster progression of complications including neuropathy and advanced‐stage DFU presentations [62, 63]. Limited access to specialised foot care for younger individuals may also delay diagnosis and treatment, resulting in more severe cases requiring hospitalisation.

Over the past two decades, Aotearoa has implemented a range of initiatives to improve diabetes DFD outcomes, including routine foot risk screening, funded community podiatry services, risk based management pathways, and multidisciplinary approaches to DFD [35]. Although these models are supported by international evidence and represent substantial progress, the increasing burden of DFD‐caused hospitalisations observed in this study indicates that significant gaps remain between policy, service provision, and outcomes. Evidence suggests that optimal implementation of foot screening and foot protection services could prevent more than 600 amputations each year and save more than $11 million in direct healthcare costs [64]. Future efforts should focus on improving equitable access to culturally safe preventive and specialist services, strengthening referral pathways, and ensuring that people identified as being at increased foot risk receive timely and appropriate care. In addition to amputation rates, DFD‐caused hospitalisations should be considered a key indicator of service performance and equity, providing a more comprehensive measure of the impact of DFD on individuals and the health system.

## 5 Limitations and Strengths

Being a single‐centre study limits the generalisability of these findings, but being Aotearoa's largest hospital provides some insights into national trends. The retrospective design, relied on existing medical records and administrative data, which may have inherent bias, but used well‐established ICD‐10(AM) codes to identify DFD‐caused hospitalisations. While associations between DFD hospitalisations and demographic factors were examined, data were unavailable for regression analysis to control for potential confounding variables, such as comorbidities or socioeconomic status. The study's 5‐year duration provided a longitudinal perspective identifying consistent trends and offering valuable insights for policymakers. Adopting the International Working Group for the DFD definition for DFD hospitalisations supported capturing the full spectrum of DFD cases and the use of the ICD‐10(AM) standardised classification systems ensured comparability of findings.

## 6 Conclusion

This is the first study in three decades to investigate DFD‐caused hospitalisations in Aotearoa, reporting significant and concerning increases in DFD‐caused hospitalisations and amputations that are placing a substantial burden on the healthcare system. Notably Māori and Pacific people experienced the highest rates of DFD‐related hospitalisations, highlighting persistent inequities in DFD outcomes. While DFD disproportionately affected males and older adults, a concerning number of hospitalisations also occurred among younger individuals. These findings support the inclusion of DFD‐caused hospitalisations rates alongside amputation rates as national quality indicators and highlight the need for strengthened, equitable, and culturally responsive foot protection services. Future efforts should focus on ensuring timely access to high‐risk foot care and establishing nationally consistent DFD prevention and management across Aotearoa.

## Author Contributions

**Michele Garrett:** conceptualization, methodology, formal analysis, writing – original draft, writing –review and editing. **Rinki Murphy:** conceptualization, writing – review and editing. **Arier Lee:** methodology, formal analysis, writing – review and editing. **Peter A. Lazzarini:** methodology, writing – review and editing. **Timothy Kenealy:** conceptualization, methodology, formal analysis, writing – review and editing.

## Conflicts of Interest

The authors declare no conflicts of interest.

## Supporting information


Supporting Information S1

## Data Availability

Data available on request due to privacy/ethical restrictions; The data that support the findings of this study are available on request from the corresponding author. The data are not publicly available due to privacy or ethical restrictions.
