# Supplementary material for: Diabetes‐Related Foot Disease as the Principal Reason for Hospital Admission: 5 Years of Data From a Major Tertiary Hospital in Aotearoa New Zealand
Source: J Foot Ankle Res. 2026 Oct 17;19(4):e70225. doi: 10.1002/jfa2.70225 (PMC13676419; doi:10.1002/jfa2.70225)
Supplement: Supplementary file 1 [file JFA2-19-e70225-s001.docx]

Supplementary material

## Appendix 1 Diabetic Foot Codes

Diabetes and diabetes foot disease definition by ICD-10(AM) codes

|  | | ICD code | Code descriptor | Other diagnosis code required to identify diabetes |
| --- | --- | --- | --- | --- |
| Diabetes |  | ICD code | E10* Type 1 DM  E11* Type 2DM  E13* Other specified DM  E14* Unspecified DM |  |
| Acute Diabetic Foot | Foot ulcer  Infection  Sepsis  Pressure injury  Gangrene  Charcot foot | E1073 E1173  E1373  E1473 | Type 1 DM with foot ulcer due to multiple causes  Type 2 DM with foot ulcer due to multiple causes  Other specified DM with foot ulcer due to multiple causes  Unspecified DM with foot ulcer due to multiple causes | None |
|  |  | L970 | Ulcer of foot | At least one of E10- E14 |
|  |  | I7023 | Atherosclerosis of arteries of extremities with ulceration | At least one of E10- E14 |
|  |  | L0243 | Cutaneous abscess, furuncle and carbuncle of toe/foot | At least one of E10- E14 |
|  |  | L0302 | Cellulitis of toe | At least one of E10- E14 |
|  |  | L0314 | Cellulitis of foot | At least one of E10- E14 |
|  |  | M8617 | Other acute osteomyelitis, ankle and foot | At least one of E10- E14 |
|  |  | M8627 | Subacute osteomyelitis, ankle and foot | At least one of E10- E14 |
|  |  | M8647 | Chronic osteomyelitis with draining sinus, ankle and foot | At least one of E10- E14 |
|  |  | M8667 | Other chronic osteomyelitis, ankle and foot | At least one of E10- E14 |
|  |  | M8687 | Other osteomyelitis, ankle and foot | At least one of E10- E14 |
|  |  | M8697 | Unspecified osteomyelitis, ankle and foot | At least one of E10- E14 |
|  |  | A400 | Sepsis due to streptococcus, group A | At least one code indicating diabetic foot ulcer (E10.73, E11.73, E13.73, E14.73)  **OR**  At least one of one code indicating diabetes (E10- E14) **TOGETHER WITH** at least one code indicating foot ulcer or infection (L970, L89*, I7023, L0243, L0302, L0314, M8617, M8627, M8667, M8687, M8697) |
|  |  | A401 | Sepsis due to streptococcus, group B |  |
|  |  | A402 | Sepsis due to streptococcus, group D and enterococcus |  |
|  |  | A403 | Sepsis due to Streptococcus pneumoniae |  |
|  |  | A408 | Other streptococcal sepsis |  |
|  |  | A409 | Streptococcal sepsis, unspecified |  |
|  |  | A410 | Sepsis due to Staphylococcus aureus |  |
|  |  | A411 | Sepsis due to other specified staphylococcus |  |
|  |  | A412 | Sepsis due to unspecified staphylococcus |  |
|  |  | A413 | Sepsis due to Haemophilus influenzae |  |
|  |  | A414 | Sepsis due to anaerobes |  |
|  |  | A4150 | Sepsis due to unspecified Gram-negative organisms |  |
|  |  | A4151 | Sepsis due to Escherichia coli [E. Coli] |  |
|  |  | A4152 | Sepsis due to Pseudomonas |  |
|  |  | A4158 | Sepsis due to other Gram-negative organisms |  |
|  |  | A418 | Other specified sepsis |  |
|  |  | A419 | Sepsis, unspecified |  |
|  |  | A499 | Bacterial infection, unspecified |  |
|  |  | L8917-19 | Pressure injury of foot, stage ll | At least one of E10- E14 |
|  |  | L8927-29 | Pressure injury of foot, stage III | At least one of E10- E14 |
|  |  | L8937-39 | Pressure injury of foot, stage IV | At least one of E10- E14 |
|  |  | L8947-49 | Pressure injury of foot, unstageable | At least one of E10- E14 |
|  |  | L8997-99 | Pressure injury, unspecified stage, ankle and foot | At least one of E10- E14 |
|  |  | E1052 E1152  E1352  E1452 | Diabetes mellitus with peripheral angiopathy, with gangrene | None |
|  |  | I7024 | Atherosclerosis of arteries of extremities with gangrene | At least one of E10-14 |
|  |  | R02 | Gangrene not classified elsewhere | At least one of E10-14 |
|  |  | E10.61 E11.61  E13.61  E14.61 | Diabetes mellitus with Charcot's arthropathy // DM with diabetic osteopathy | None |
